# Supplementary material for: PheWAS and cross-disorder analysis reveal genetic architecture, pleiotropic loci and phenotypic correlations across 11 autoimmune disorders
Source: Front Immunol. 2023 Sep 21;14:1147573. doi: 10.3389/fimmu.2023.1147573 (PMC10552152; doi:10.3389/fimmu.2023.1147573)
Supplement: Supplementary file 3 [file DataSheet_1.pdf]

Supplement to "PheWAS and cross-disorder analyses reveal genetic architecture, pleiotropic loci and phenotypic correlations across 11 autoimmune disorders."

Topaloudi et al.

Figure S1 Overview of the phenotypes included in the general UK Biobank categories. 2
Figure S2 Overview of the phenotypes included in the general UK Biobank categories. 3
Figure S3: Percentage of phenotypes associated with autoimmune polygenic risk scores (p<10-5). 4
Figure S4 Volcano plot of all PheWAS results for each autoimmune disorder. 10
Figure S5 Significant PRS-PheWAS for at least three AD PRS with phenotypes in the Health and Medical History UK Biobank category, using the normalized PRS. 11
Figure S6 Significant PRS-PheWAS for at least three AD PRS with phenotypes in the Sociodemographics UK Biobank category, using the normalized PRS. 13
Figure S7 Significant PRS-PheWAS for at least three AD PRS with phenotypes in the Biomarkers (Blood Count) UK Biobank category, using the normalized PRS. 14
Figure S8 Significant PRS-PheWAS for at least three AD PRS with phenotypes in the Biomarkers (Blood biochemistry) UK Biobank category, using the normalized PRS. 16
Figure S9 Significant PRS-PheWAS for at least three AD PRS with phenotypes in the Biomarkers (Infectious Diseases) UK Biobank category, using the normalized PRS. 18
Figure S10 Significant PRS-PheWAS for at least three AD PRS with phenotypes in the Physical Measures UK Biobank category, using the normalized PRS. 19
Figure S11 Genetic correlation of the 11 autoimmune disorders. 20
Figure S12 Significant PRS-PheWAS for all AD PRS in factor one with the phenotypes in UK Biobank. 21
Figure S13 Significant PRS-PheWAS for all AD PRS in factor two with the phenotypes in UK Biobank. 22
Figure S14 Significant PRS-PheWAS for all AD PRS in factor three with the phenotypes in UK Biobank. 23
Figure S15 Significant PRS-PheWAS for all AD PRS in factor four with the phenotypes in UK Biobank. 24

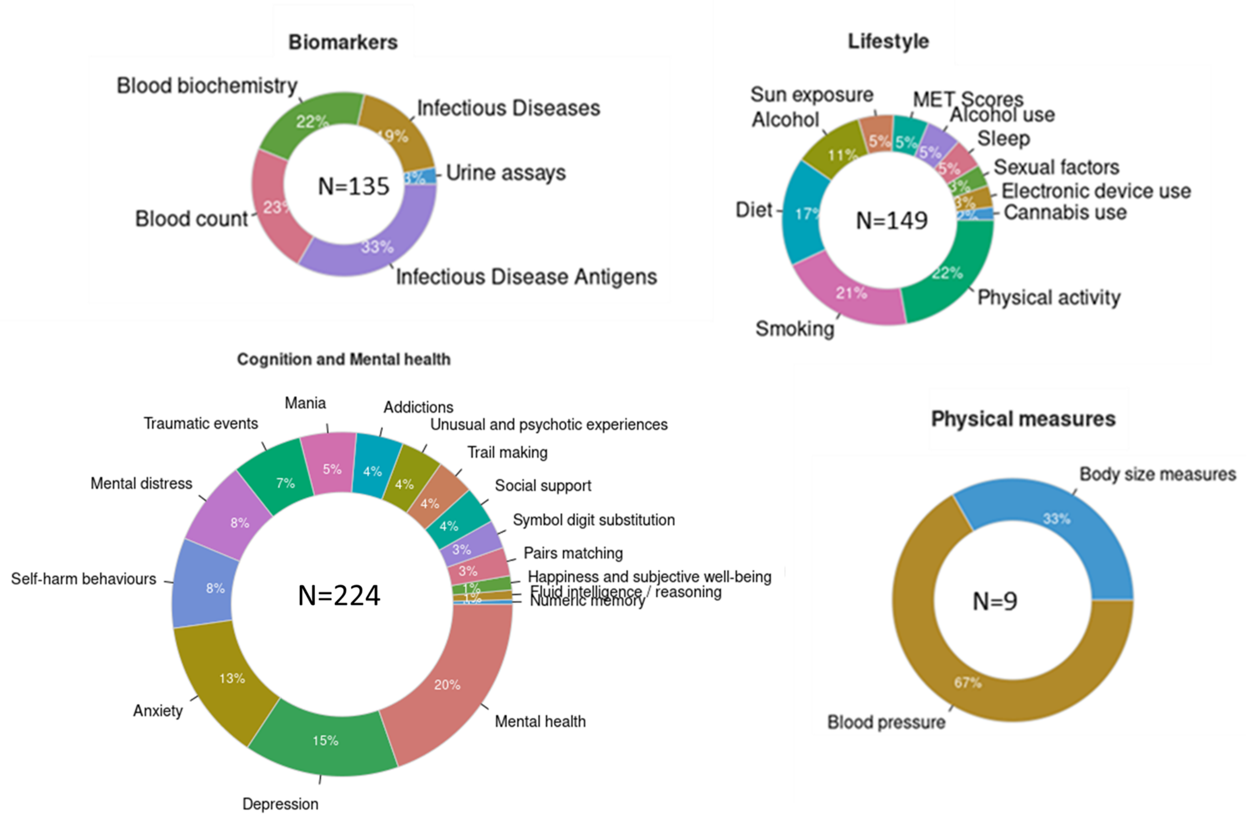

Figure S1 Overview of the phenotypes included in the general UK Biobank categories.
The number inside the circles shows the total number of phenotypes in each category. The numbers in each block show the percentage of phenotypes included in each sub-category.

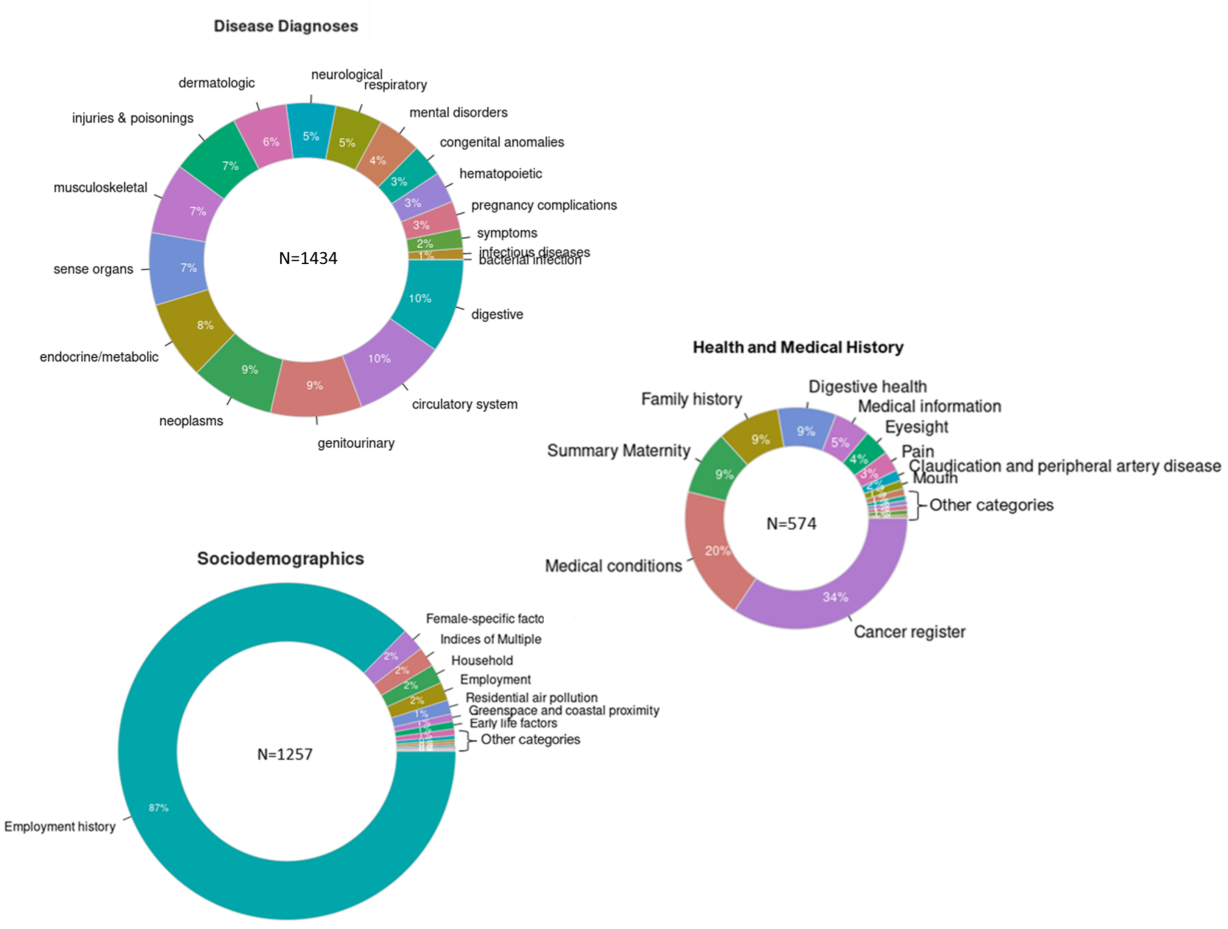

Figure S2 Overview of the phenotypes included in the general UK Biobank categories.
The number inside the circles shows the total number of phenotypes in each category. The numbers in each block show the percentage of phenotypes included in each sub-category.

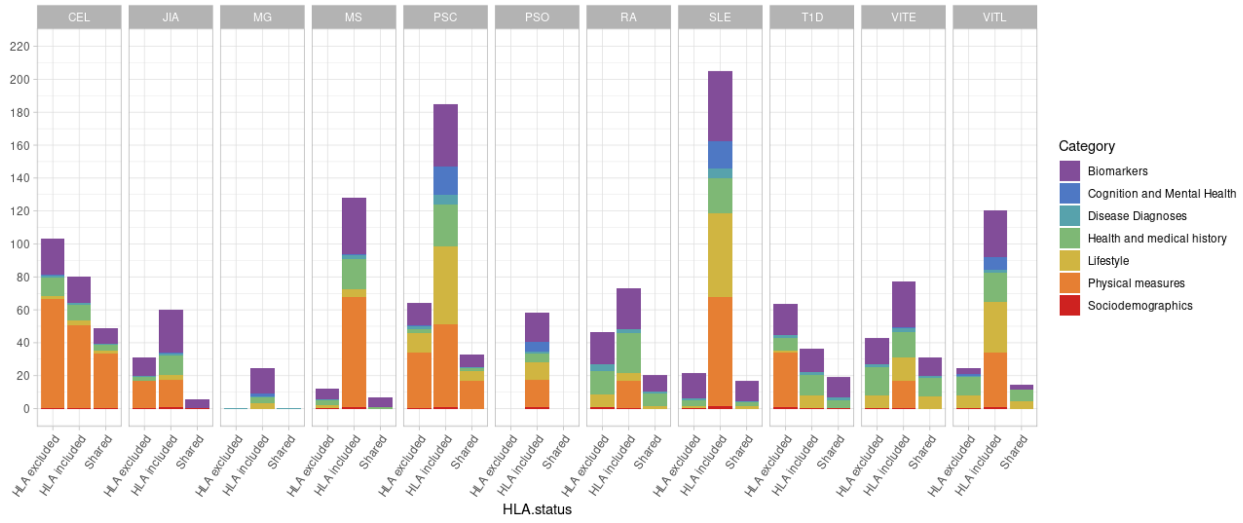

Figure S3: Percentage of phenotypes associated with autoimmune polygenic risk scores ($p<10^{-5}$). The different colors represent the general UK Biobank categories. The "HLA excluded" bar shows the percentage of the significant associations with the phenotypes when HLA was excluded from the AD PRS calculations. The "HLA included" bar shows the percentage of the significant associations with the phenotypes when HLA was included in the AD PRS calculations. The "Shared" bar shows the percentage of the significant associations with the phenotypes for both HLA included or excluded AD PRSs.

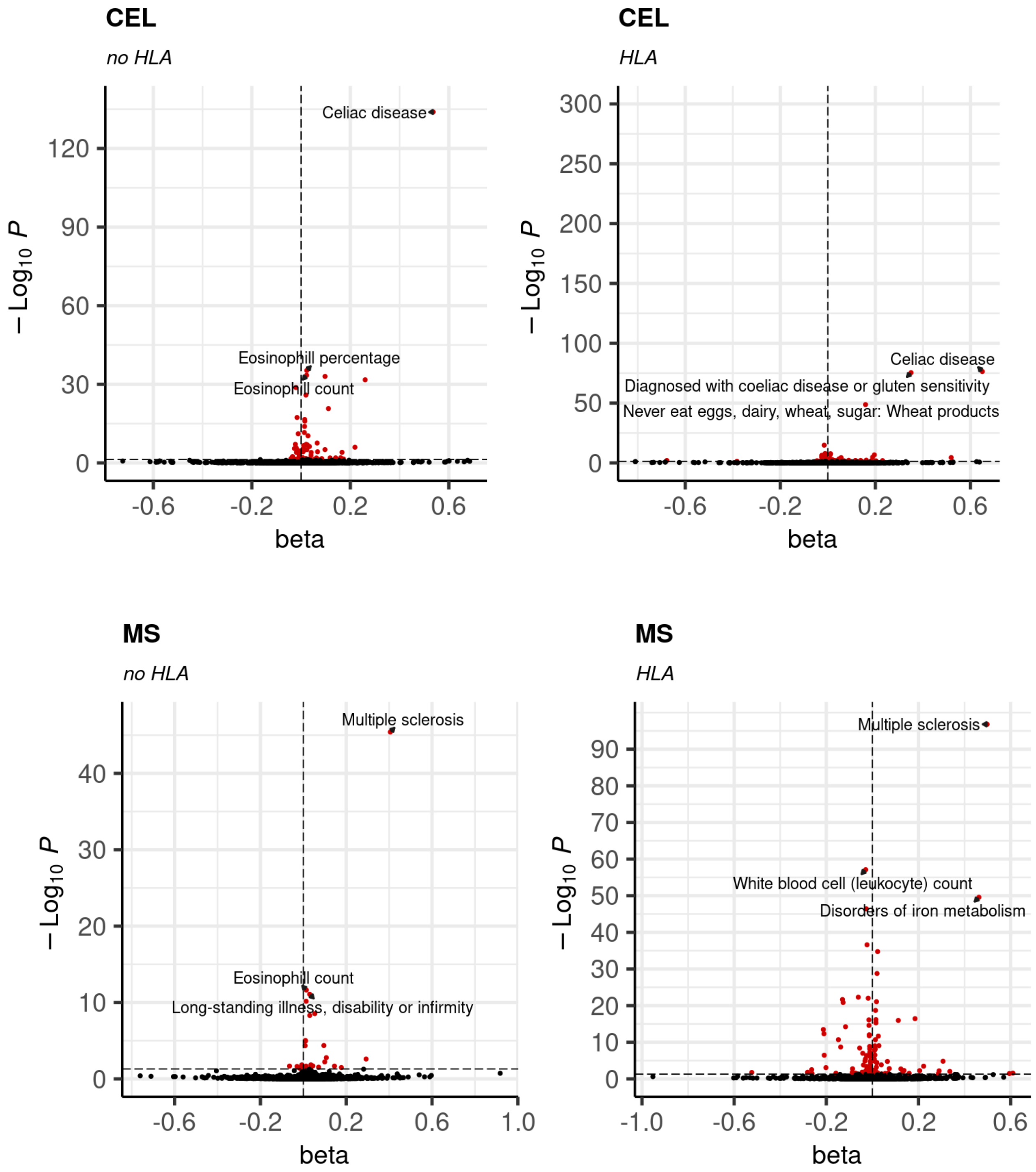

CEL
no HLA
Celiac disease
Eosinophill percentage
Eosinophill count
− Log10 P
beta
CEL
HLA
Celiac disease
Diagnosed with coeliac disease or gluten sensitivity
Never eat eggs, dairy, wheat, sugar: Wheat products
− Log10 P
beta
MS
no HLA
Multiple sclerosis
Eosinophill count
Long-standing illness, disability or infirmity
− Log10 P
beta
MS
HLA
Multiple sclerosis
White blood cell (leukocyte) count
Disorders of iron metabolism
− Log10 P
beta

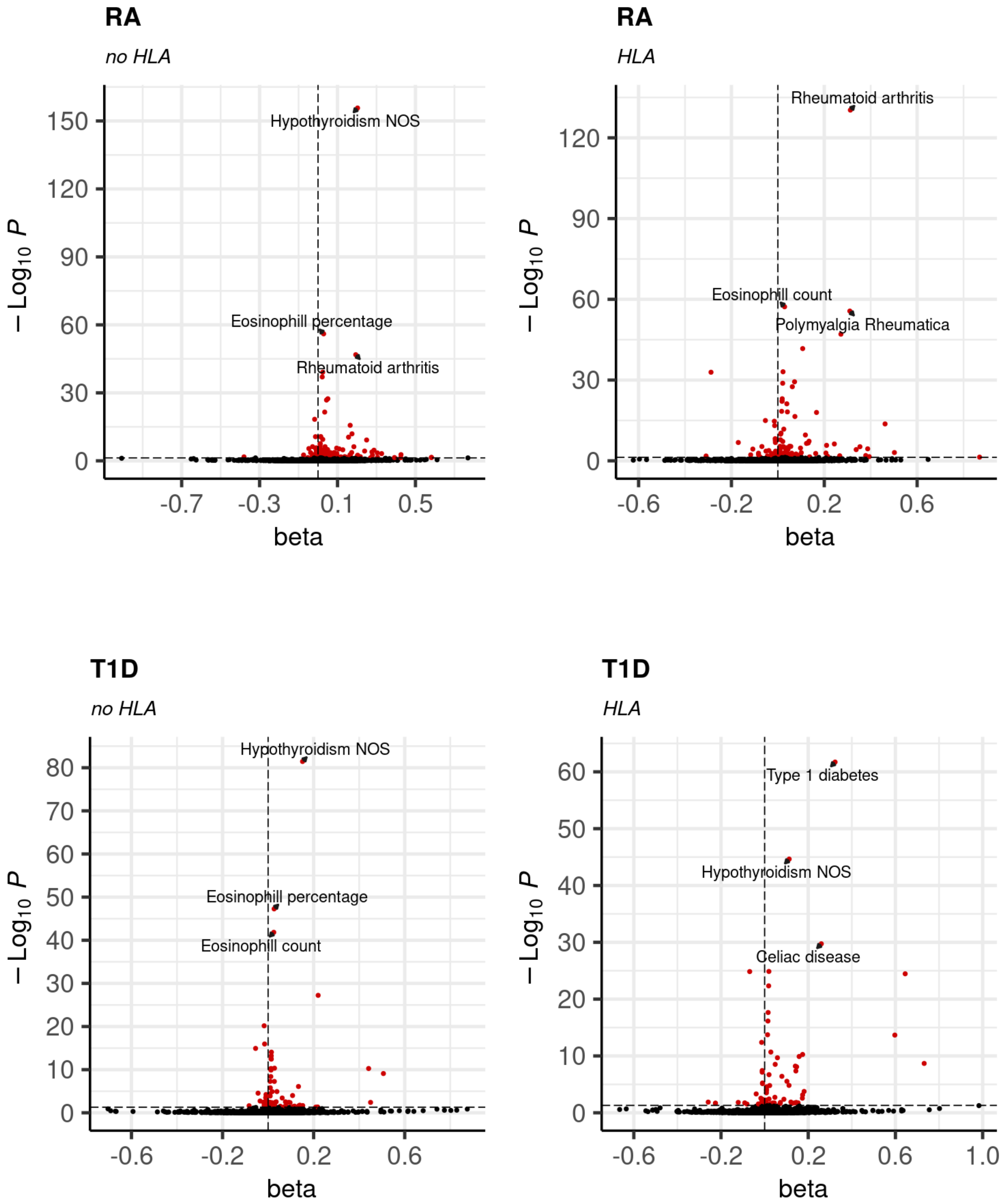
RA
no HLA
Hypothyroidism NOS
Eosinophill percentage
Rheumatoid arthritis
150
120
90
60
30
0
-0.7
-0.3
0.1
0.5
beta
RA
HLA
Rheumatoid arthritis
Eosinophill count
Polymyalgia Rheumatica
120
90
60
30
0
-0.6
-0.2
0.2
0.6
beta
T1D
no HLA
Hypothyroidism NOS
Eosinophill percentage
Eosinophill count
80
70
60
50
40
30
20
10
0
-0.6
-0.2
0.2
0.6
beta
T1D
HLA
Type 1 diabetes
Hypothyroidism NOS
Celiac disease
60
50
40
30
20
10
0
-0.6
-0.2
0.2
0.6
1.0
beta

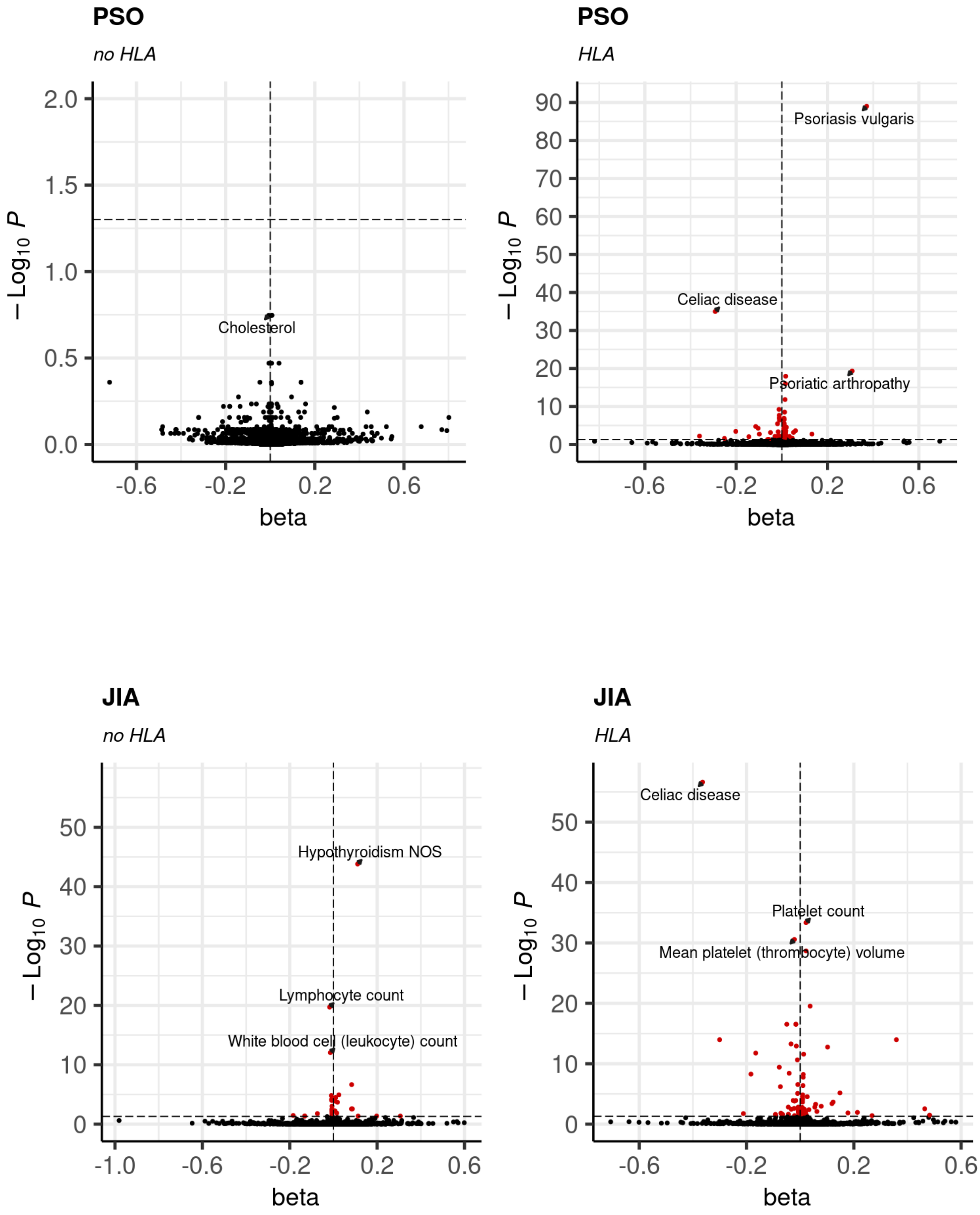
PSO
no HLA
Cholesterol
2.0
1.5
1.0
0.5
0.0
−Log10 P
-0.6
-0.2
0.2
0.6
beta
PSO
HLA
Psoriasis vulgaris
Celiac disease
Psoriatic arthropathy
90
80
70
60
50
40
30
20
10
0
−Log10 P
-0.6
-0.2
0.2
0.6
beta
JIA
no HLA
Hypothyroidism NOS
Lymphocyte count
White blood cell (leukocyte) count
50
40
30
20
10
0
−Log10 P
-1.0
-0.6
-0.2
0.2
0.6
beta
JIA
HLA
Celiac disease
Platelet count
Mean platelet (thrombocyte) volume
50
40
30
20
10
0
−Log10 P
-0.6
-0.2
0.2
0.6
beta

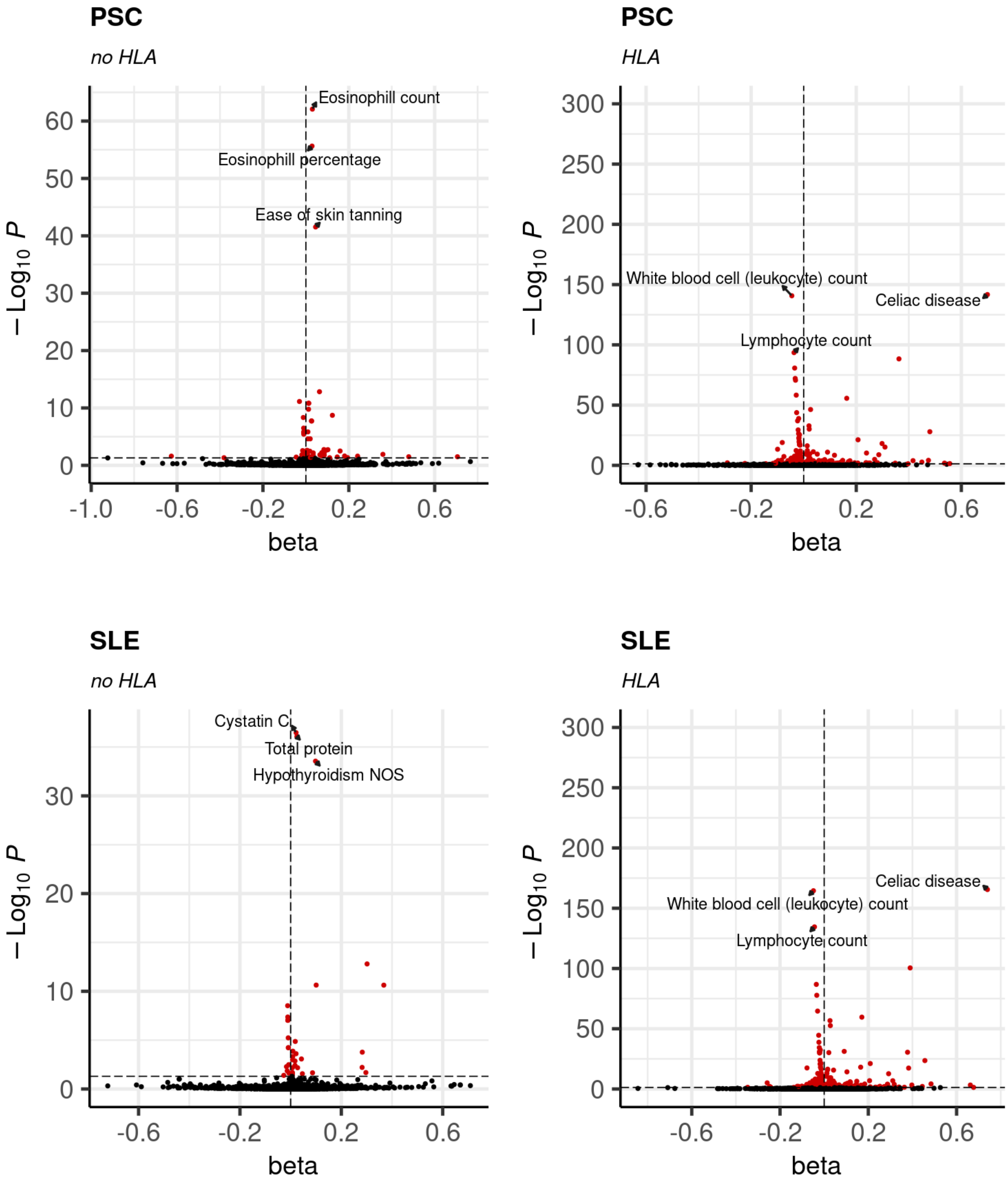
PSC
no HLA
Eosinophill count
Eosinophill percentage
Ease of skin tanning
− Log10 P
beta
PSC
HLA
White blood cell (leukocyte) count
Celiac disease
Lymphocyte count
− Log10 P
beta
SLE
no HLA
Cystatin C
Total protein
Hypothyroidism NOS
− Log10 P
beta
SLE
HLA
Celiac disease
White blood cell (leukocyte) count
Lymphocyte count
− Log10 P
beta

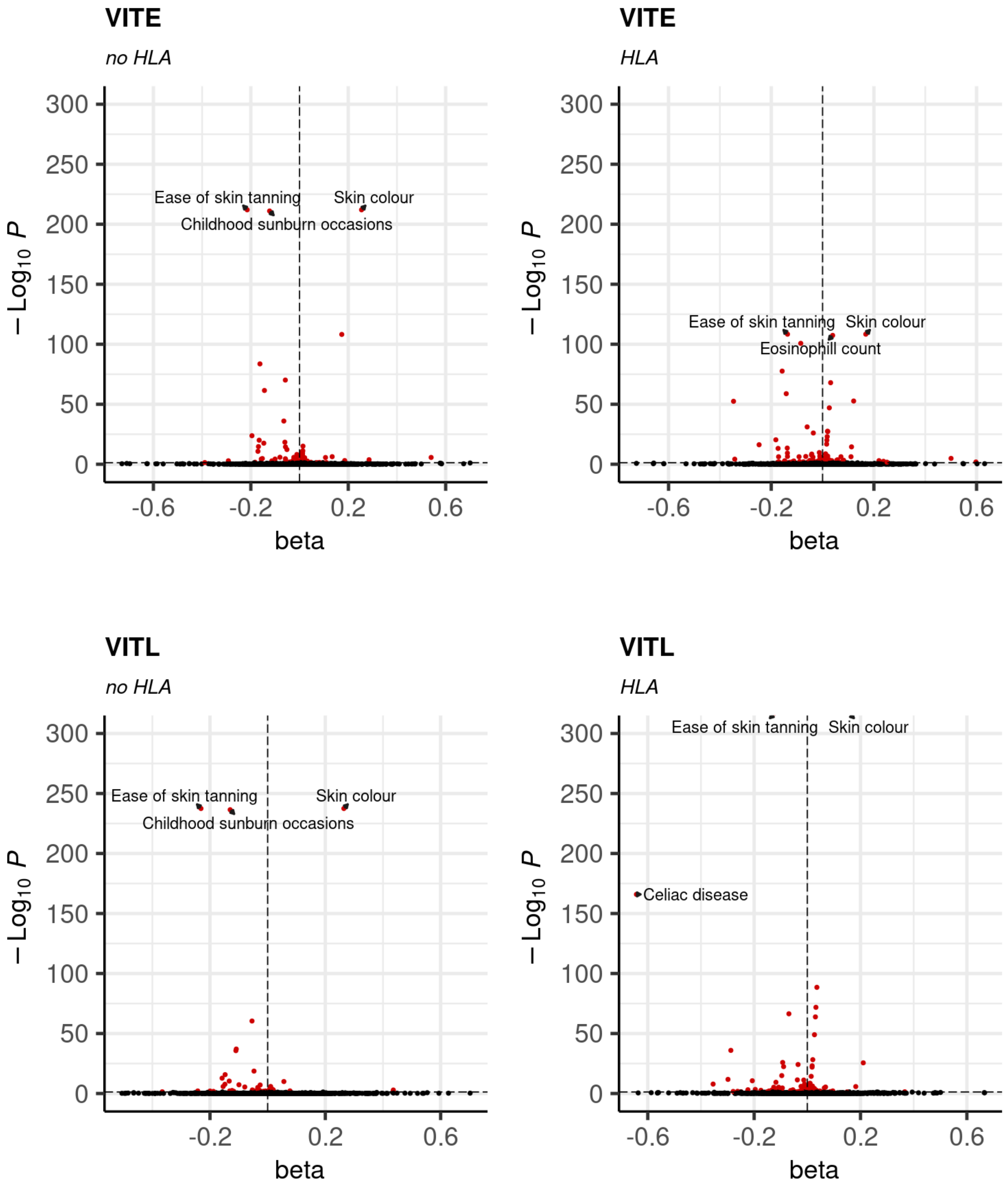

VITE
no HLA
Ease of skin tanning
Skin colour
Childhood sunburn occasions
300
250
200
150
100
50
0
-0.6
-0.2
0.2
0.6
beta
− Log10 P
VITE
HLA
Ease of skin tanning
Skin colour
Eosinophill count
beta
VITL
no HLA
Ease of skin tanning
Skin colour
Childhood sunburn occasions
beta
VITL
HLA
Ease of skin tanning
Skin colour
Celiac disease
beta

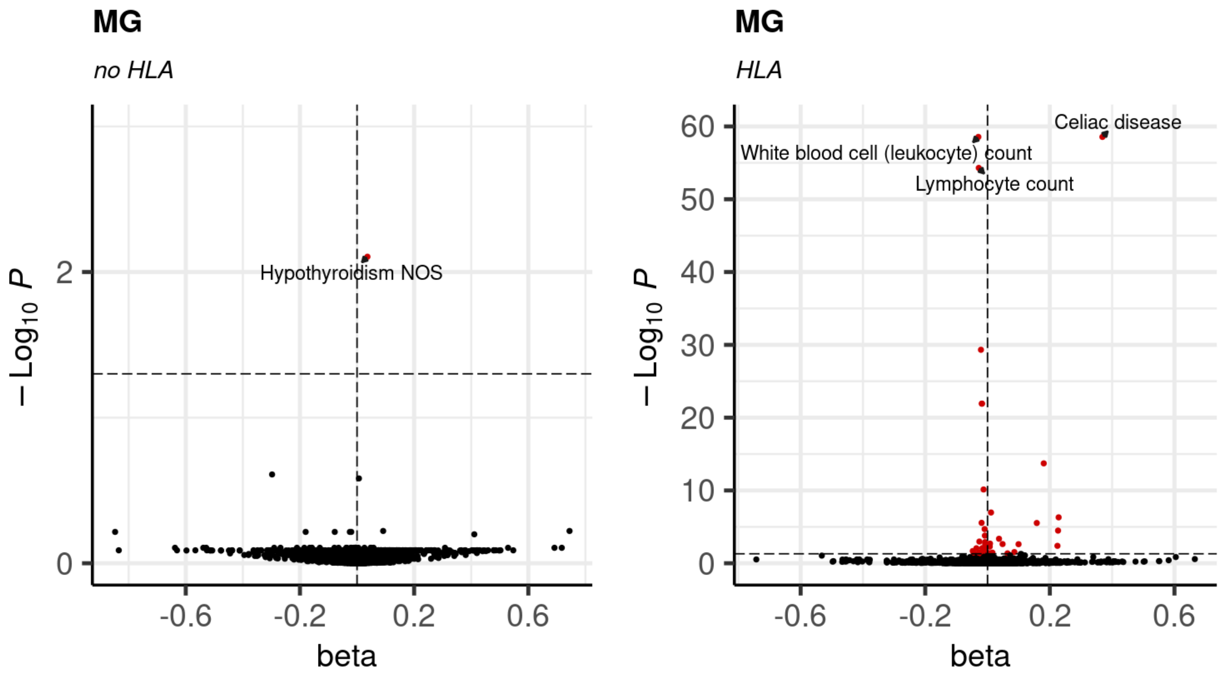

Figure S4 Volcano plot of all PheWAS results for each autoimmune disorder.
Annotated are the top 3 significant results, and with red the outcomes with pFDR<0.05.

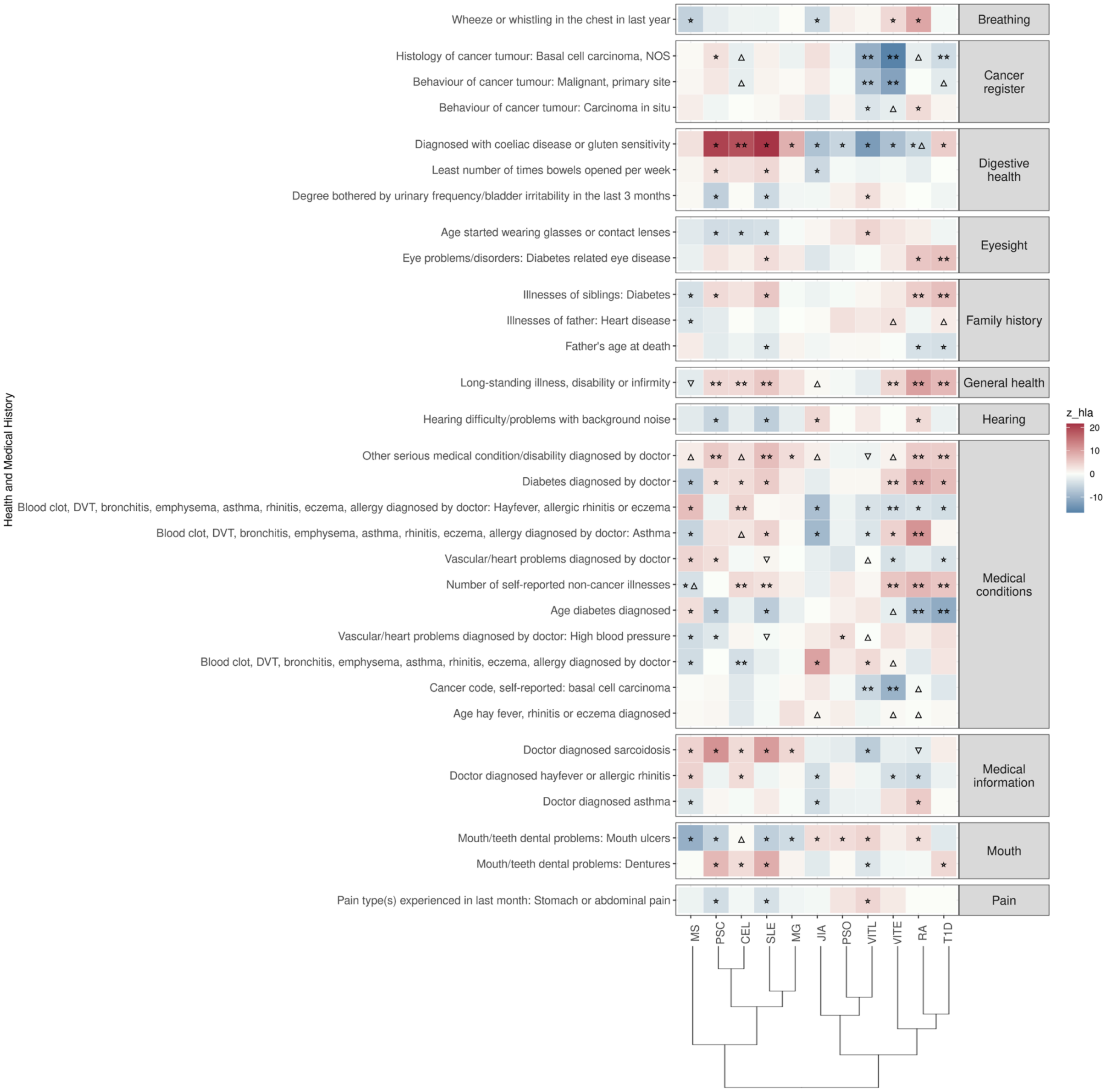

Figure S5 Significant PRS-PheWAS for at least three AD PRS with phenotypes in the Health and Medical History UK Biobank category, using the normalized PRS.

The shown phenotypes were significantly associated, after FDR adjustment, with at least three AD PRS irrespectively of the HLA status. The colors of cells indicate the standardized effect sizes (β) for the regression between AD PRS with HLA and each phenotype. The one star "☆" shows the significant results only with the "HLA included" AD PRS. The two stars "☆☆" show the significant associations with both "HLA included or excluded" AD PRS with the same effect direction. The star and the upper facing triangle "☆▵" show the significant associations with both "HLA included or excluded" AD PRS but with opposite effect directions. The upper facing triangle "▵" shows the significant associations only

with “HLA excluded” AD PRS that the effect direction is the same as the color indicates. For the grouping of the phenotypes, we used the categories provided by the UK Biobank. We used the hclust R function to perform the hierarchical clustering of the autoimmune disorders showing in the dendrogram using all standardized effect sizes for the health and medical history category phenotypes.

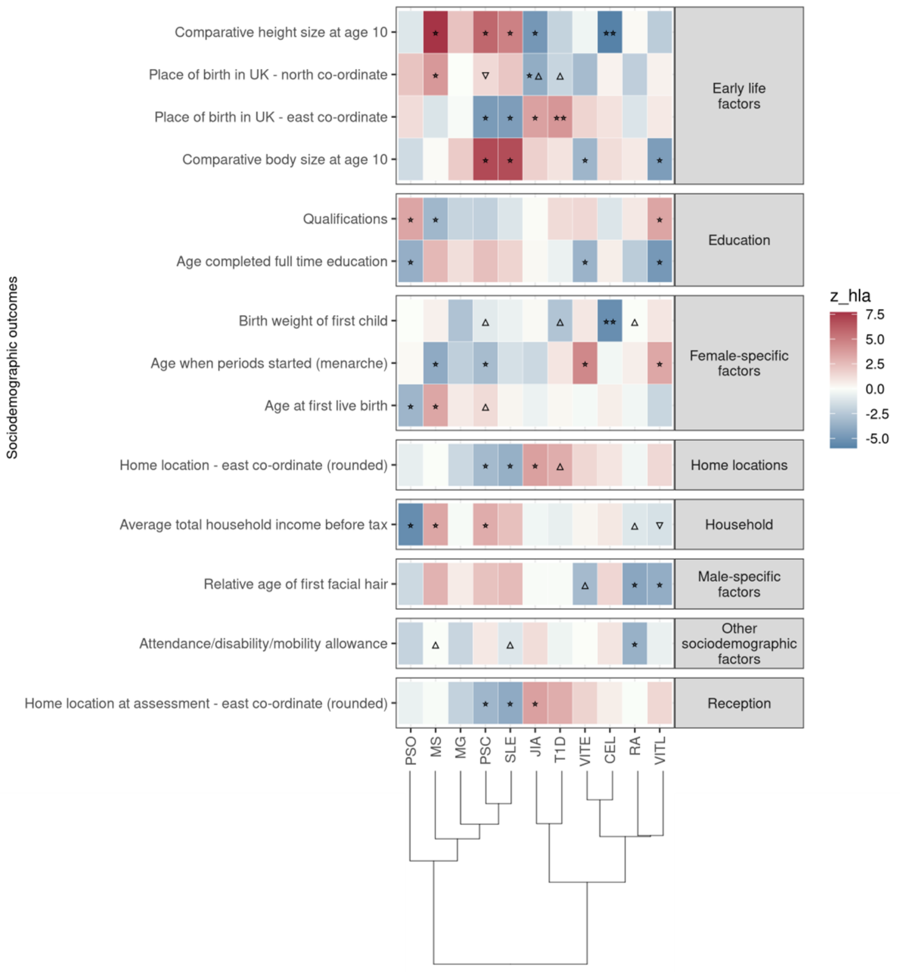

Figure S6 Significant PRS-PheWAS for at least three AD PRS with phenotypes in the Sociodemographics UK Biobank category, using the normalized PRS.

The shown phenotypes were significantly associated, after FDR adjustment, with at least three AD PRS irrespectively of the HLA status. The colors of cells indicate the standardized effect sizes (β) for the regression between AD PRS with HLA and each phenotype. The one star "☆" shows the significant results only with the "HLA included" AD PRS. The two stars "☆☆" show the significant associations with both "HLA included or excluded" AD PRS with the same effect direction. The star and the upper facing triangle "☆▵" show the significant associations with both "HLA included or excluded" AD PRS but with opposite effect directions. The upper facing triangle "▵" shows the significant associations only with "HLA excluded" AD PRS that the effect direction is the same as the color indicates. The down-facing triangle "▿" shows the significant associations only with "HLA excluded" AD PRS that the effect direction is the opposite of what the color indicates.For the grouping of the phenotypes, we used the categories provided by the UK Biobank. We used the hclust R function to perform the hierarchical clustering of the autoimmune disorders showing in the dendrogram using all standardized effect sizes for the Sociodemographics category phenotypes.

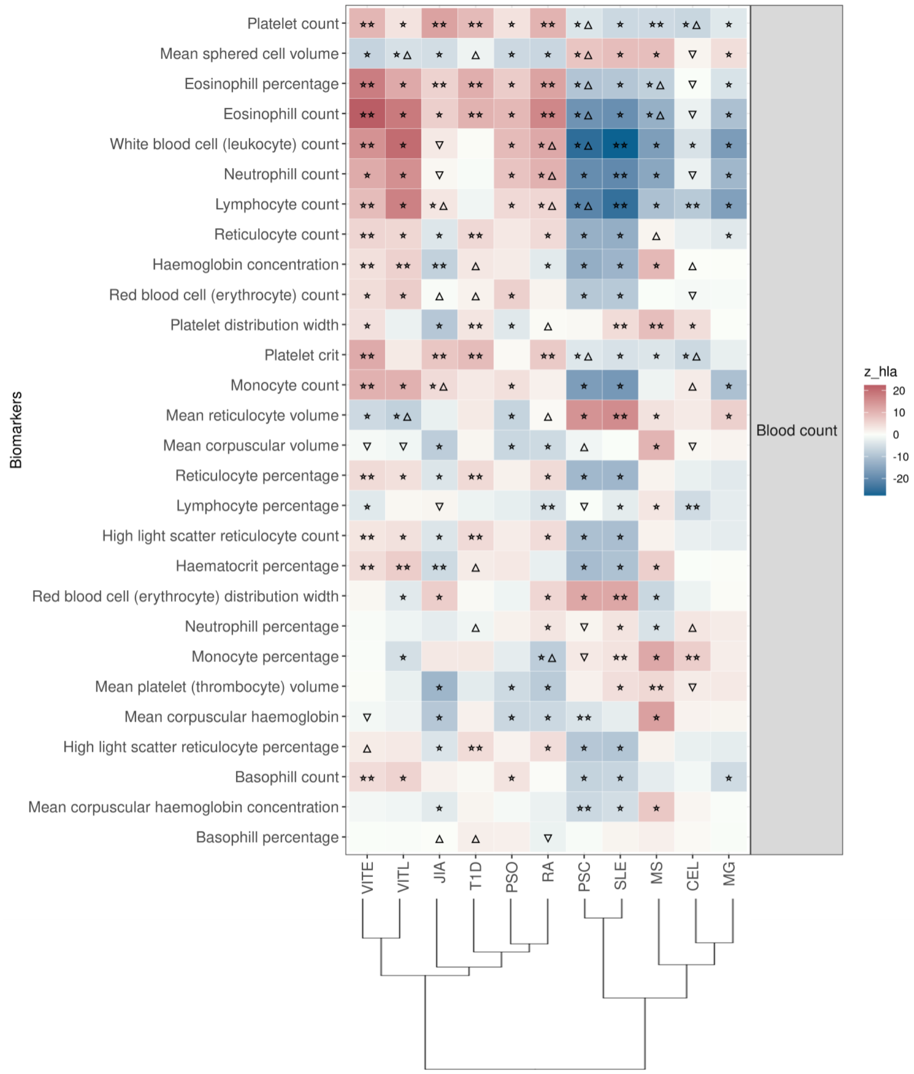

Figure S7 Significant PRS-PheWAS for at least three AD PRS with phenotypes in the Biomarkers (Blood Count) UK Biobank category, using the normalized PRS.

The shown phenotypes were significantly associated, after FDR adjustment, with at least three AD PRS irrespectively of the HLA status. The colors of cells indicate the standardized effect sizes (β) for the regression between AD PRS with HLA and each phenotype. The one star "☆" shows the significant results only with the "HLA included" AD PRS. The two stars "☆☆" show the significant associations with both "HLA included or excluded" AD PRS with the same effect direction. The star and the upper facing triangle "☆▵" show the significant associations with both "HLA included or excluded" AD PRS but with opposite effect directions. The upper facing triangle "▵" shows the significant associations only with "HLA excluded" AD PRS that the effect direction is the same as the color indicates. The down-facing triangle "▿" shows significant associations only with "HLA excluded" AD PRS that the effect direction is the opposite of what the color indicates. For the grouping of the phenotypes, we used the categories provided by the UK Biobank. We used the hclust R function to perform the

hierarchical clustering of the autoimmune disorders showing in the dendrogram using all standardized effect sizes for the Biomarkers category phenotypes.

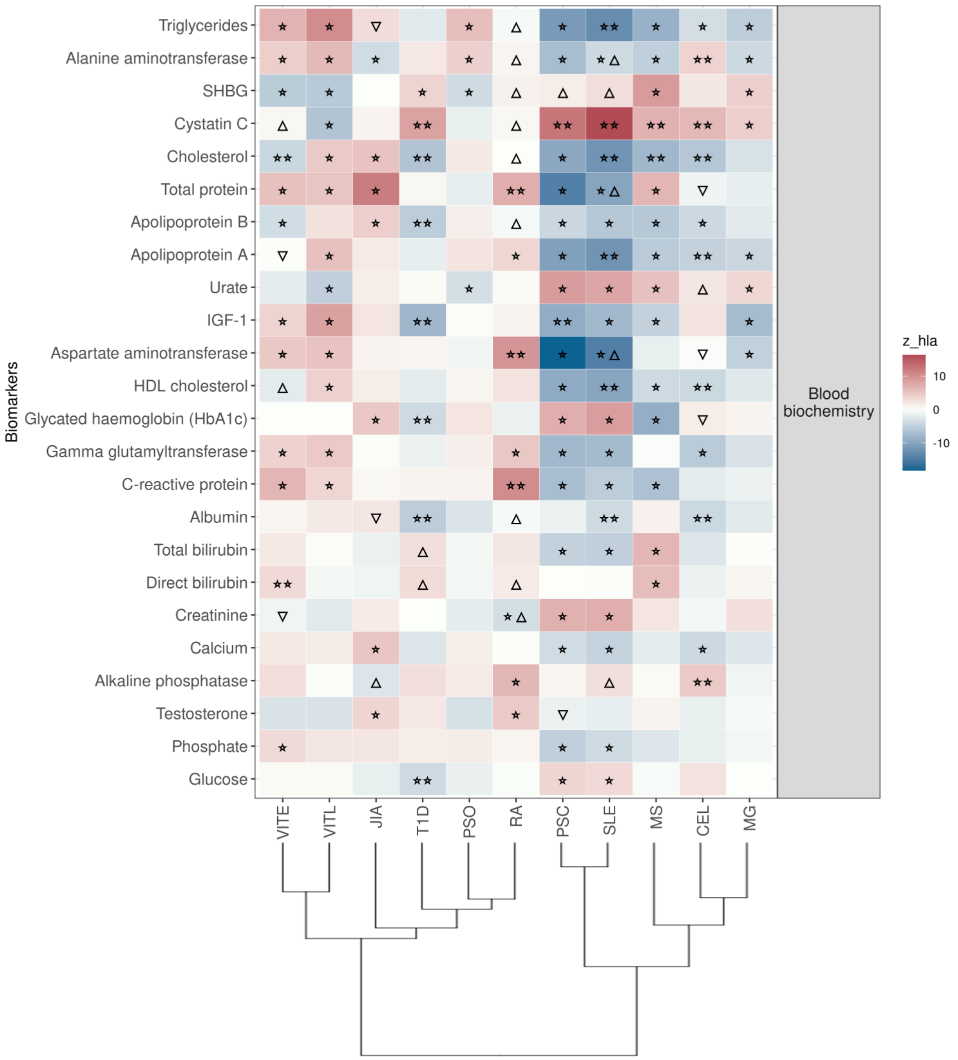

Figure S8 Significant PRS-PheWAS for at least three AD PRS with phenotypes in the Biomarkers (Blood biochemistry) UK Biobank category, using the normalized PRS.

The shown phenotypes were significantly associated, after FDR adjustment, with at least three AD PRS irrespectively of the HLA status. The colors of cells indicate the standardized effect sizes (β) for the regression between AD PRS with HLA and each phenotype. The one star "☆" shows the significant results only with the "HLA included" AD PRS. The two stars "☆☆" show the significant associations with both "HLA included or excluded" AD PRS with the same effect direction. The star and the upper facing triangle "☆▵" show the significant associations with both "HLA included or excluded" AD PRS but with opposite effect directions. The upper facing triangle "▵" shows the significant associations only with "HLA excluded" AD PRS that the effect direction is the same as the color indicates. The down-facing triangle "▿" shows significant associations only with "HLA excluded" AD PRS that the effect direction is the opposite of what the color indicates. For the grouping of the phenotypes, we used the categories provided by the UK Biobank. We used the hclust R function to perform the

hierarchical clustering of the autoimmune disorders showing in the dendrogram using all standardized effect sizes for the Biomarkers category phenotypes.

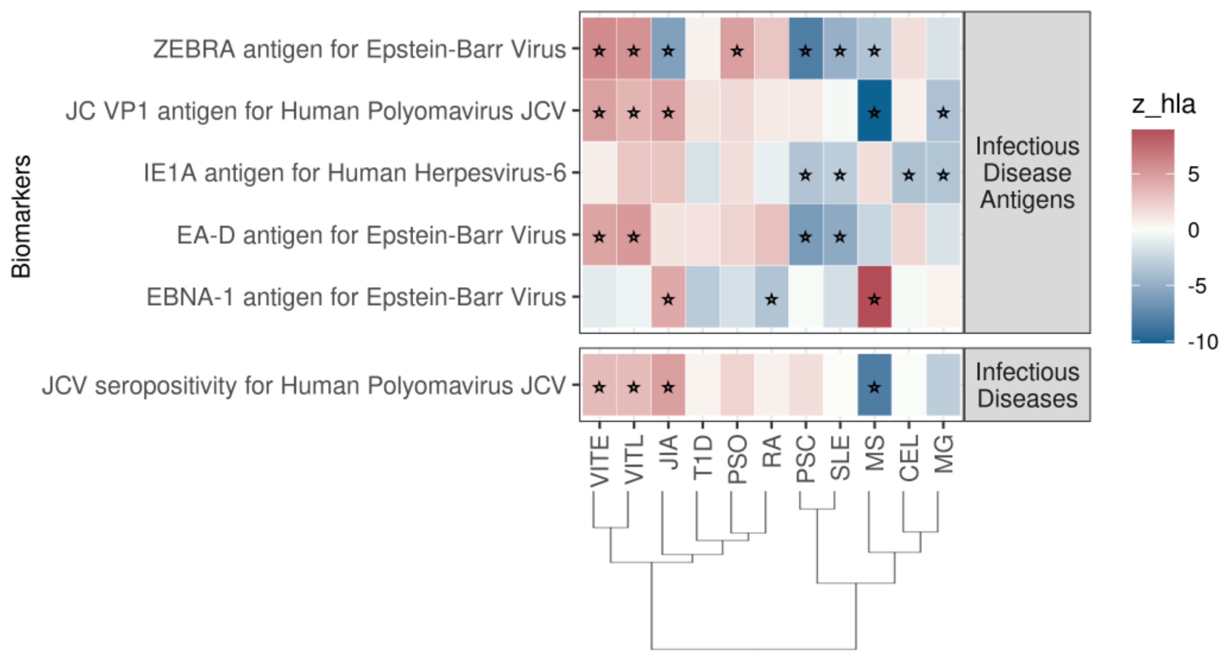

Figure S9 Significant PRS-PheWAS for at least three AD PRS with phenotypes in the Biomarkers (Infectious Diseases) UK Biobank category, using the normalized PRS.
The shown phenotypes were significantly associated, after FDR adjustment, with at least three AD PRS irrespectively of the HLA status. The colors of cells indicate the standardized effect sizes (β) for the regression between AD PRS with HLA and each phenotype. The one star "☆" shows the significant results only with the "HLA included" AD PRS. For the grouping of the phenotypes, we used the categories provided by the UK Biobank. We used the hclust R function to perform the hierarchical clustering of the autoimmune disorders showing in the dendrogram using all standardized effect sizes for the Biomarkers category phenotypes.

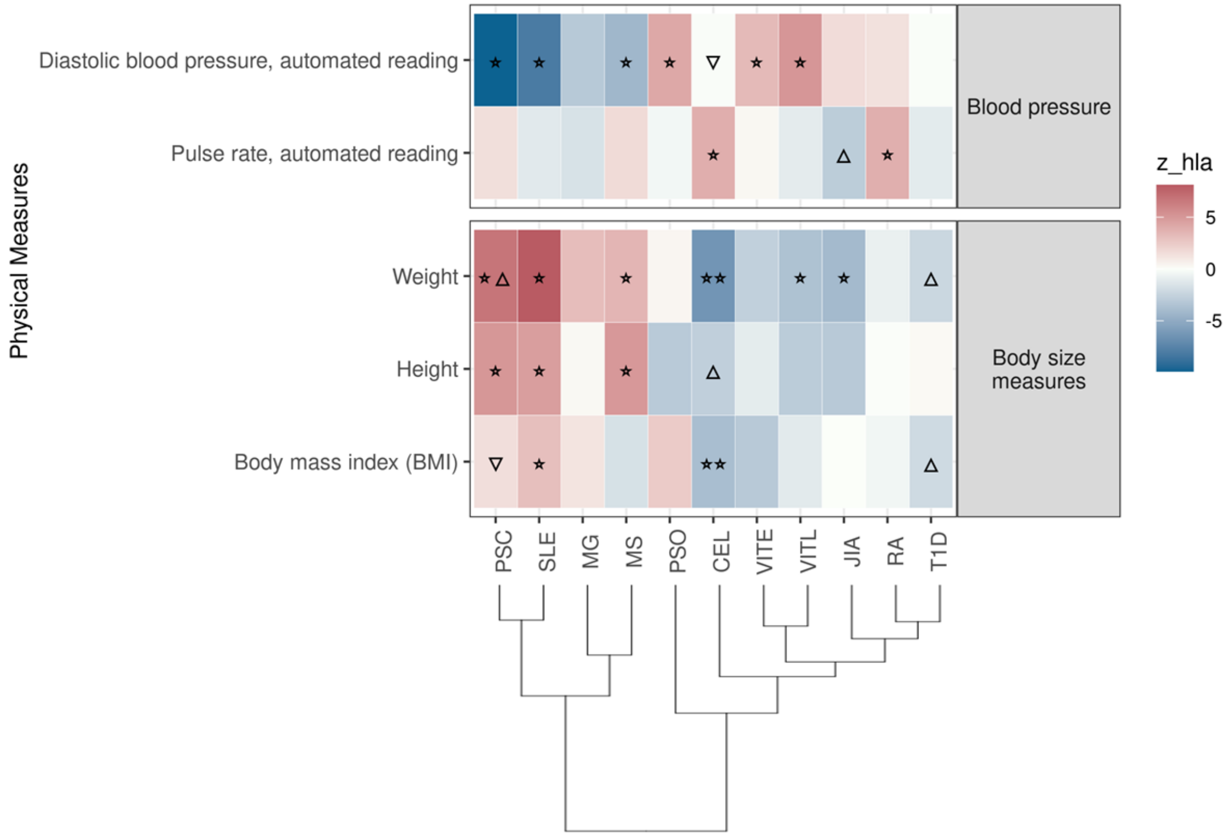

Figure S10 Significant PRS-PheWAS for at least three AD PRS with phenotypes in the Physical Measures UK Biobank category, using the normalized PRS.

The shown phenotypes were significantly associated, after FDR adjustment, with at least three AD PRS irrespectively of the HLA status. The colors of cells indicate the standardized effect sizes (β) for the regression between AD PRS with HLA and each phenotype. The one star "☆" shows the significant results only with the "HLA included" AD PRS. The two stars "☆☆" show the significant associations with both "HLA included or excluded" AD PRS with the same effect direction. The star and the upper facing triangle "☆▵" show the significant associations with both "HLA included or excluded" AD PRS but with opposite effect directions. The upper facing triangle "▵" shows the significant associations only with "HLA excluded" AD PRS that the effect direction is the same as the color indicates. The down-facing triangle "▿" shows the significant associations only with "HLA excluded" AD PRS that the effect direction is the opposite of what the color indicates. For the grouping of the phenotypes, we used the categories provided by the UK Biobank. We used the hclust R function to perform the hierarchical clustering of the autoimmune disorders showing in the dendrogram using all standardized effect sizes for the Physical Measures category phenotypes.

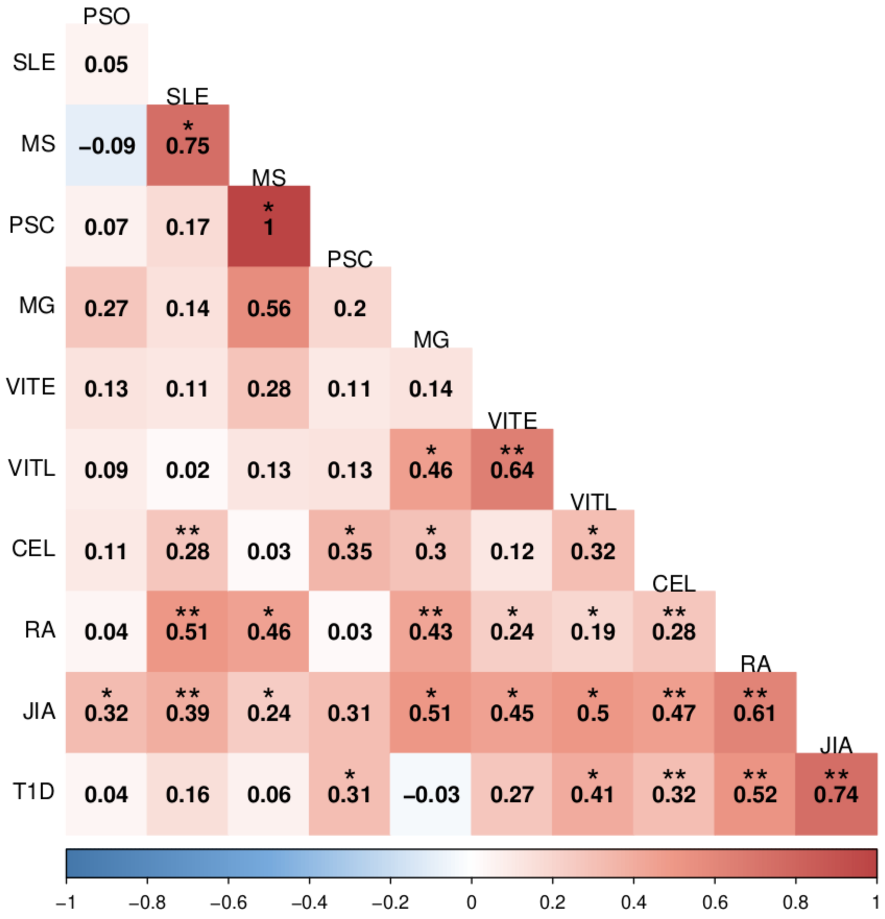

Figure S11 Genetic correlation of the 11 autoimmune disorders.
The figure shows the genetic correlation for all 11 autoimmune disorders included in the analysis. A) After excluding the SNPs in the HLA region (hg19, chr6 25-33 Mb). B) Without excluding the SNPs in the HLA region. The red color in the cells reflects more positive correlation coefficients while blue reflects more negative coefficients, and the numbers within each cell are the correlation coefficients. The correlations with p<0.05 are denoted with one asterisk (*), while the two asterisks show the correlations that are significant after the Bonferroni correction.

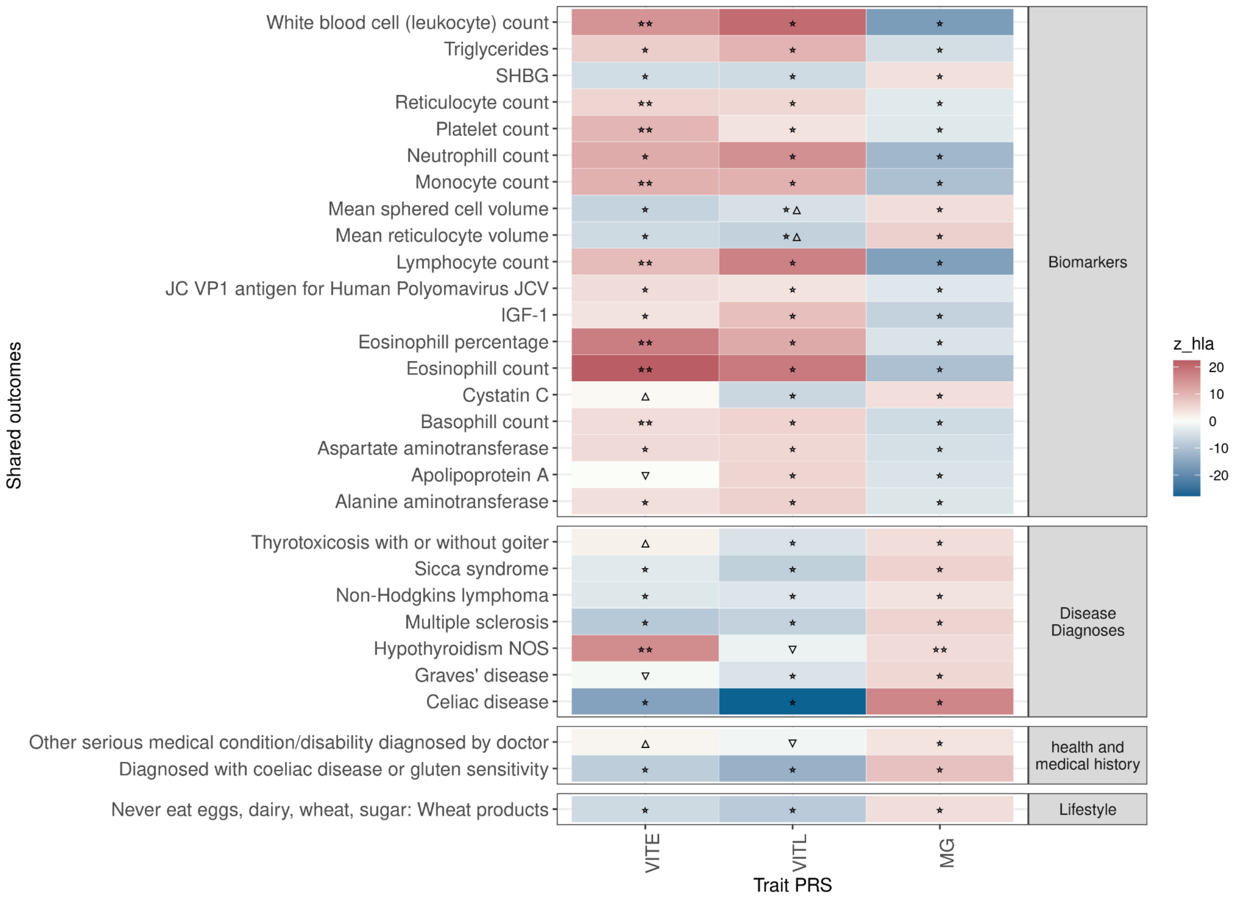

Figure S12 Significant PRS-PheWAS for all AD PRS in factor one with the phenotypes in UK Biobank.

The shown phenotypes were significantly associated, after FDR adjustment, with at all AD PRS in factor one irrespectively of the HLA status. The colors of cells indicate the standardized effect sizes (β) for the regression between AD PRS with HLA and each phenotype. The one star "☆" shows the significant results only with the "HLA included" AD PRS. The two stars "☆☆" show the significant associations with both "HLA included or excluded" AD PRS with the same effect direction. The star and the upper facing triangle "☆△" show the significant associations with both "HLA included or excluded" AD PRS but with opposite effect directions. The upper facing triangle "△" shows the significant associations only with "HLA excluded" AD PRS that the effect direction is the same as the color indicates. The down-facing triangle "▽" shows the significant associations only with "HLA excluded" AD PRS that the effect direction is the opposite of what the color indicates.

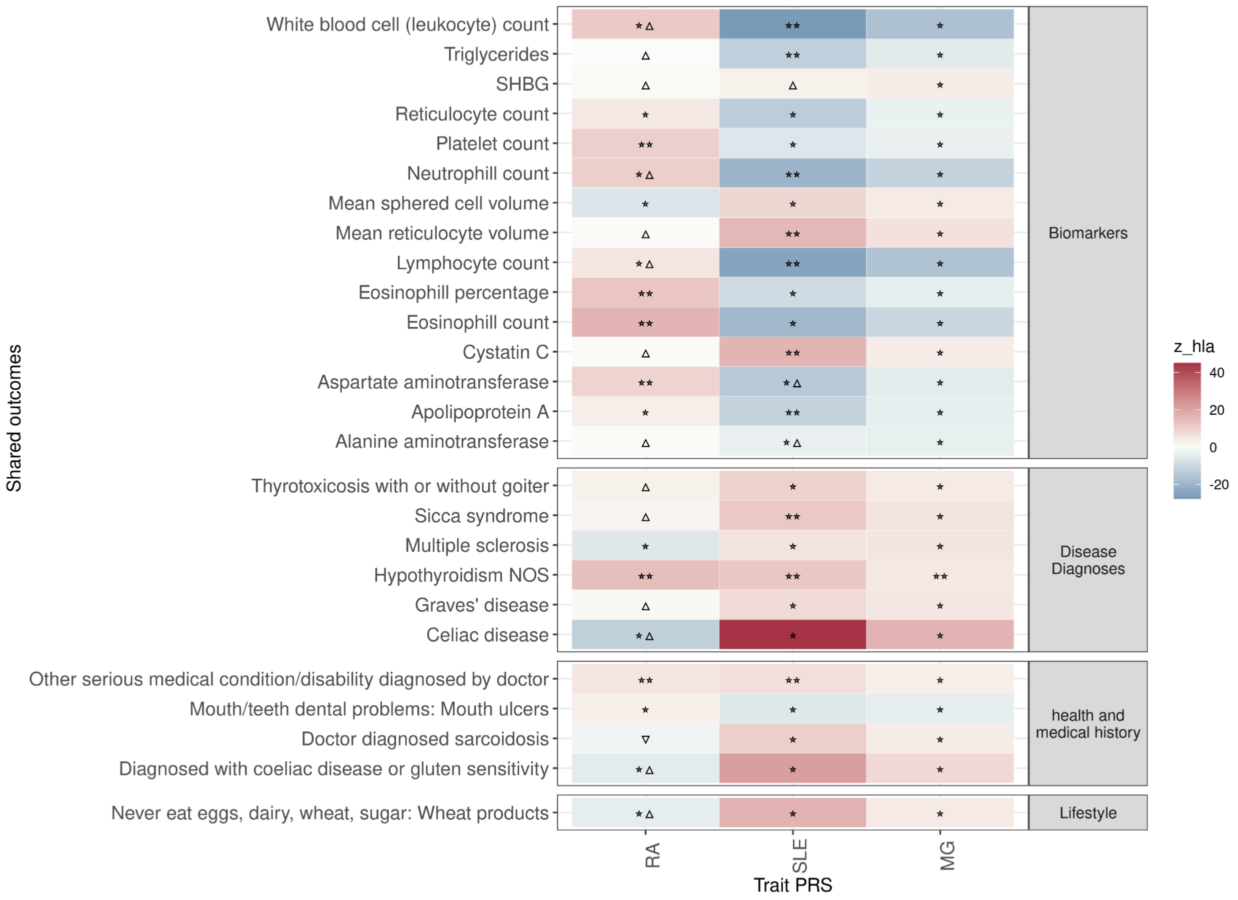

Figure S13 Significant PRS-PheWAS for all AD PRS in factor two with the phenotypes in UK Biobank.

The shown phenotypes were significantly associated, after FDR adjustment, with at all AD PRS in factor two irrespectively of the HLA status. The colors of cells indicate the standardized effect sizes (β) for the regression between AD PRS with HLA and each phenotype. The one star "☆" shows the significant results only with the "HLA included" AD PRS. The two stars "☆☆" show the significant associations with both "HLA included or excluded" AD PRS with the same effect direction. The star and the upper facing triangle "☆▵" show the significant associations with both "HLA included or excluded" AD PRS but with opposite effect directions. The upper facing triangle "▵" shows the significant associations only with "HLA excluded" AD PRS that the effect direction is the same as the color indicates. The down-facing triangle "▿" shows the significant associations only with "HLA excluded" AD PRS that the effect direction is the opposite of what the color indicates.

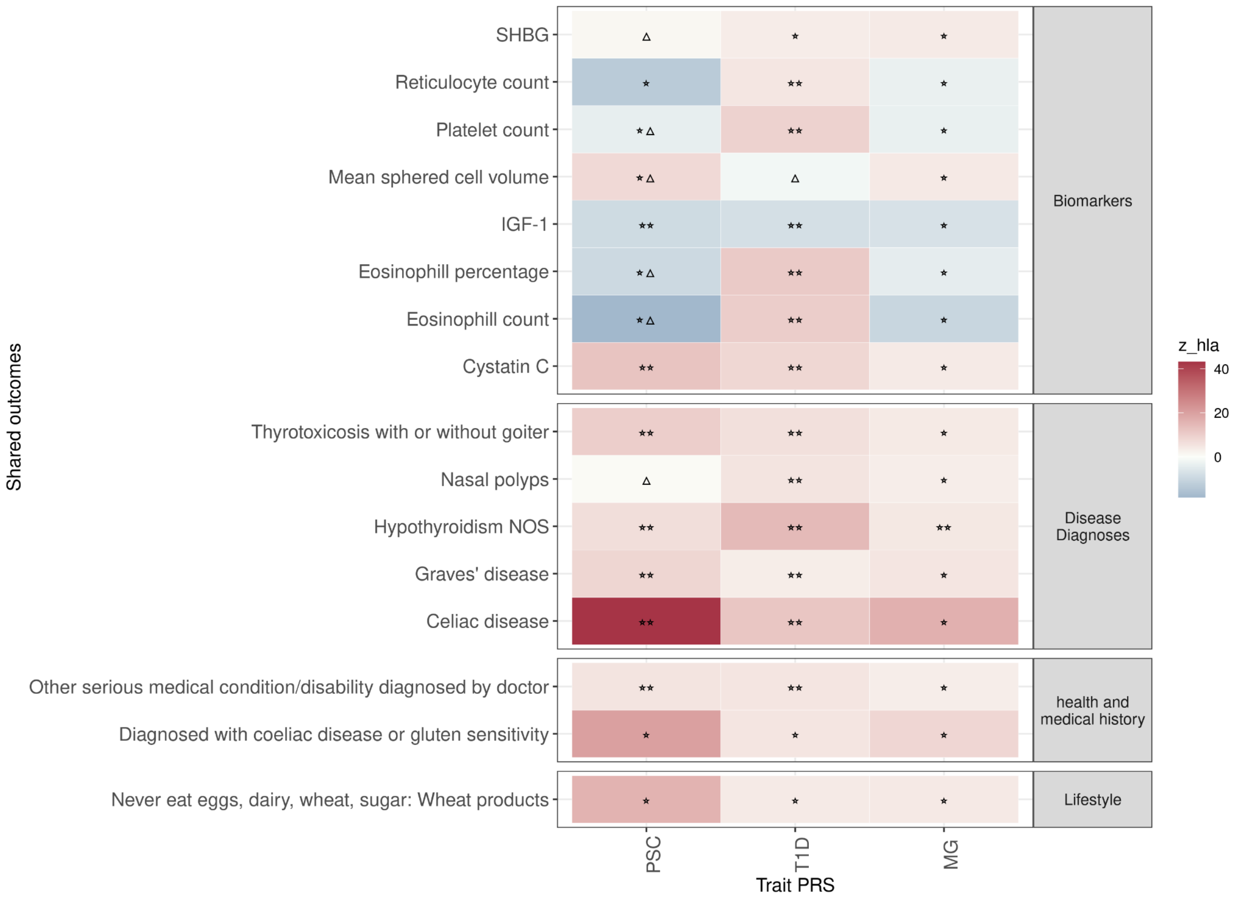

Figure S14 Significant PRS-PheWAS for all AD PRS in factor three with the phenotypes in UK Biobank.

The shown phenotypes were significantly associated, after FDR adjustment, with at all AD PRS in factor three irrespectively of the HLA status. The colors of cells indicate the standardized effect sizes (β) for the regression between AD PRS with HLA and each phenotype. The one star "☆" shows the significant results only with the "HLA included" AD PRS. The two stars "☆☆" show the significant associations with both "HLA included or excluded" AD PRS with the same effect direction. The star and the upper facing triangle "☆△" show the significant associations with both "HLA included or excluded" AD PRS but with opposite effect directions. The upper facing triangle "△" shows the significant associations only with "HLA excluded" AD PRS that the effect direction is the same as the color indicates. The down-facing triangle "▽" shows the significant associations only with "HLA excluded" AD PRS that the effect direction is the opposite of what the color indicates.

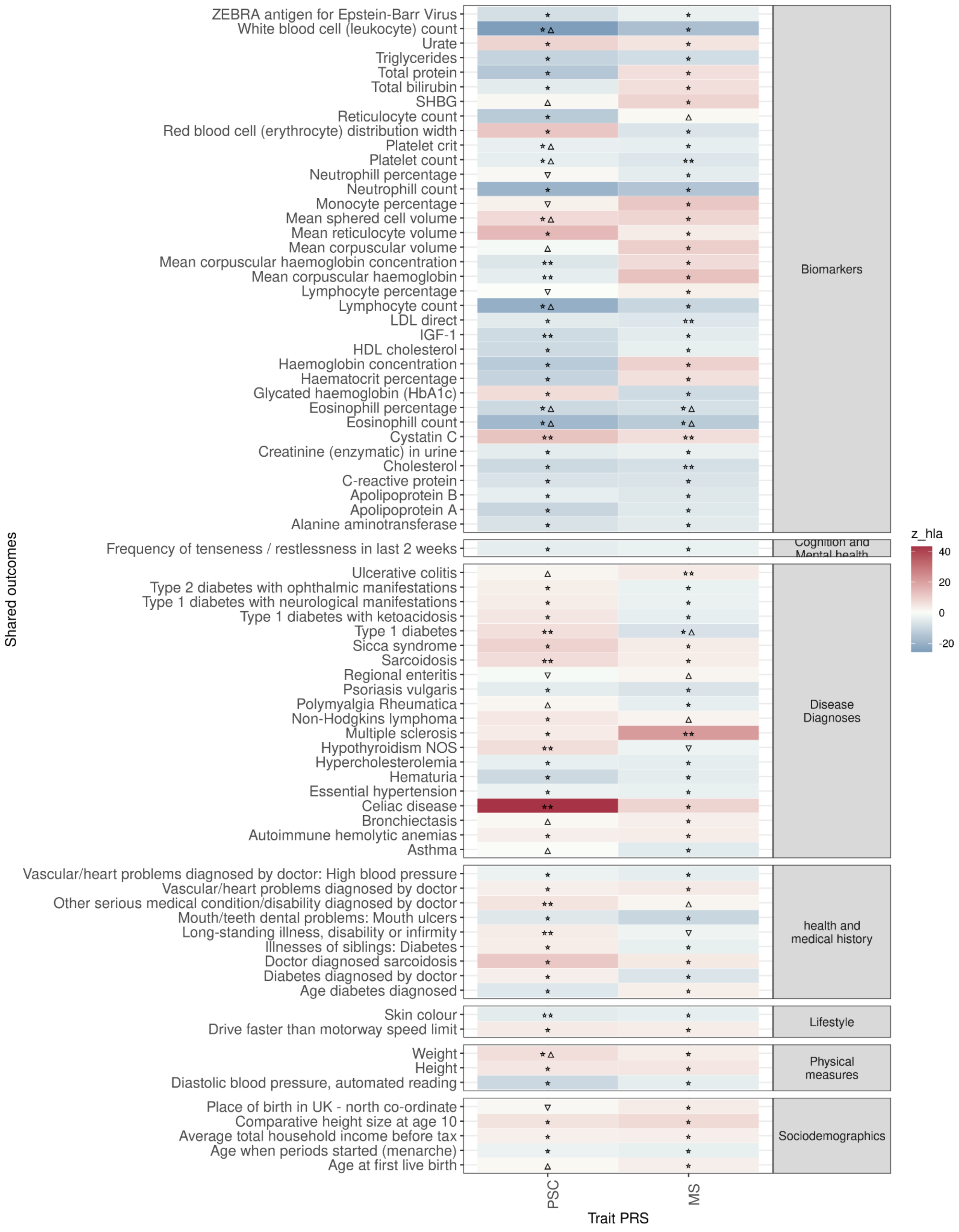

Figure S15 Significant PRS-PheWAS for all AD PRS in factor four with the phenotypes in UK Biobank.

The shown phenotypes were significantly associated, after FDR adjustment, with at all AD PRS in factor four irrespectively of the HLA status. The colors of cells indicate the standardized effect sizes (β) for the regression between AD PRS with HLA and each phenotype. The one star "☆" shows the significant results only with the "HLA included" AD PRS. The two stars "☆☆" show the significant associations with both "HLA included or excluded" AD PRS with the same effect direction. The star and the upper facing triangle "☆△" show the significant associations with both "HLA included or excluded" AD PRS but with opposite effect directions. The upper facing triangle "△" shows the significant associations only with "HLA excluded" AD PRS that the effect direction is the same as the color indicates. The down-facing triangle "▽" shows the significant associations only with "HLA excluded" AD PRS that the effect direction is the opposite of what the color indicates.
